# Short-Term Repeated Wingate Training in Hypoxia and Normoxia in Sprinters

**DOI:** 10.3389/fspor.2020.00043

**Published:** 2020-04-22

**Authors:** Naoya Takei, Katsuyuki Kakinoki, Olivier Girard, Hideo Hatta

**Affiliations:** ^1^Department of Sports Sciences, The University of Tokyo, Tokyo, Japan; ^2^Murdoch Applied Sports Science Laboratory, Murdoch University, Perth, WA, Australia; ^3^Blue Wych Limited Company, Kanagawa, Japan; ^4^School of Human Sciences (Exercise and Sport Science), The University of Western Australia, Crawley, WA, Australia

**Keywords:** altitude training, hypoxia, live low train high, “all out” efforts, blood lactate concentration

## Abstract

Repeated Wingate efforts (RW) represent an effective training strategy for improving exercise capacity. Living low-training high altitude/hypoxic training methods, that upregulate muscle adaptations, are increasingly popular. However, the benefits of RW training in hypoxia compared to normoxia on performance and accompanying physiological adaptations remain largely undetermined. Our intention was to test the hypothesis that RW training in hypoxia provides additional performance benefits and more favorable physiological responses than equivalent training in normoxia. Twelve male runners (university sprinters) completed six RW training sessions (3 × 30-s Wingate “all-out” efforts with 4.5-min recovery) in either hypoxia (FiO_2_: 0.145, *n* = 6) or normoxia (FiO_2_: 0.209, *n* = 6) over 2 weeks. Before and after the intervention, participants underwent a RW performance test (3 × 30-s Wingate “all-out” efforts with 4.5-min recovery). Peak power output, mean power output, and total work for the three exercise bouts were determined. A capillary blood sample was taken for analyzing blood lactate concentration (BLa) 3 min after each of the three efforts. Peak power output (+ 11.3 ± 23.0%, *p* = 0.001), mean power output (+ 6.6 ± 6.8%, *p* = 0.001), and total work (+ 6.3 ± 5.4% *p* = 0.016) significantly increased from pre- to post-training, independently of condition. The time × group × interval interaction was significant (*p* = 0.05) for BLa. Compared to Pre-tests, BLa values during post-test were higher (+ 8.7 ± 10.3%) after about 2 in the normoxic group, although statistical significance was not reached (*p* = 0.08). Contrastingly, BLa values were lower (albeit not significantly) during post- compared to pre-tests after bout 2 (−9.3 ± 8.6%; *p* = 0.08) and bout 3 (−9.1 ± 10.7%; *p* = 0.09) in the hypoxic group. In conclusion, six RW training sessions over 2 weeks significantly improved RW performance, while training in hypoxia had no additional benefit over normoxia. However, accompanying BLa responses tended to be lower in the hypoxic group, while an opposite pattern was observed in the normoxic group. This indicates that different glycolytic and/or oxidative pathway adaptations were probably at play.

## Introduction

Live low-train high altitude/hypoxic training methods are increasingly popular (Wilber, 2007a; Faiss et al., 2013b; Brocherie et al., 2017). Such interventions are associated with limited costs and travel constraints for athletes who can remain in their home environment and maintain their usual lifestyle, while training a few times a week under hypoxic conditions. Due to insufficient hypoxic exposure duration, it is unlikely that living low-training high evokes beneficial hematological adaptations in the form of augmented red blood cell number or total hemoglobin mass (Wilber, 2007b). However, training in hypoxia could induce non-hematological adaptations such as upregulation in mitochondrial biogenesis (Vogt et al., 2001; Schmutz et al., 2010), oxidative and glycolytic enzymes (Vogt et al., 2001; Zoll et al., 2006; Puype et al., 2013), monocarboxylate transporters (Zoll et al., 2006; Faiss et al., 2013a) and/or angiogenesis (Vogt et al., 2001; Wahl et al., 2013). These adaptations develop in skeletal muscle tissues through an oxygen sensing signaling pathway and thereby might be less pronounced in normoxic conditions (Richardson et al., 1995; Hoppeler and Vogt, 2001). Training in hypoxia compared to normoxia could augment the exercise stimulus to boost physiological adaptations, yet it does not always provide additional performance benefits (Vogt and Hoppeler, 2010). Reduced oxygen flux resulting from lower oxygen availability can in turn negatively impact training stimulus by reducing absolute training intensity and/or volume (Vogt and Hoppeler, 2010).

Repeated Wingate efforts (RW) is a form of sprint interval training involving the repetition of “all out” 30-s efforts (Gibala et al., 2012; Maclnnis and Gibala, 2017). RW is an effective training strategy for upregulating mitochondrial biogenesis and exercise capacity (Gibala et al., 2012; Maclnnis and Gibala, 2017). Reportedly, only six sessions of RW can induce physiological and performance adaptations (Gibala et al., 2012; Maclnnis and Gibala, 2017). Hypoxic exposure can impair performance of repeated short (4–6 s efforts) sprints with short recoveries (<30-s, exercise-to-rest ratio of 1:4–1:5; Bowtell et al., 2014; Goods et al., 2014). Contrastingly, acute moderate or severe hypoxia (FiO_2_: 0.164 and 0.136) had no detrimental effect on performance of RW, presumably because longer recoveries (4-min, exercise-to-rest ratio of 1:8) would subsequently reduce stress on anaerobic mechanisms for energy restoration (Kon et al., 2015; Takei et al., 2020a). By inducing a more potent physiological stimulus, yet with preserved training quality, RW training in hypoxia would be more effective than in normoxia. In support, RW training (4–9 × 30-s Wingate efforts with 4.5-min recovery) in hypoxia (FiO_2_: 0.144) *vs*. normoxia significantly upregulated glycolytic enzyme activity, while inducing similar performance benefits (Puype et al., 2013). Whereas, first and last exercise bouts of the RW training session were “all-out” efforts, all other efforts in the study by Puype et al. (2013) were in fact completed at submaximal intensity (~80% of mean power output achieved during first sprint). Arguably, exercising at submaximal intensity would reduce training effects, with a lesser degree of muscle deoxygenation achieved during exercise periods likely attenuating stimulation of the O_2_ sensing signaling pathway (Richardson et al., 1995; Hoppeler and Vogt, 2001).

Whereas a higher glycolytic energy production is required early during RW exercise, aerobic energy production (i.e., lactate oxidation by mitochondria) plays a more prominent role to meet the energy demand during later exercise parts (Parolin et al., 1999). Although chronic hypoxic exposure may have detrimental effects on mitochondrial adaptations, repeated continuous but brief exposure to hypoxia (e.g., hypoxic training sessions) can improve mitochondrial density (Hoppeler et al., 2003). RW training in hypoxia compared to normoxia may maximize lactate utilization due to improved mitochondrial density, as indirectly assessed by blood lactate (BLa) response. To our knowledge, no previous study has examined the effects of RW training in hypoxia on the time course of BLa responses following each effort forming a RW exercise.

Therefore, our intention was to test hypothesis that RW training (3 × 30-s Wingate “all-out” efforts with 4.5-min recovery) in moderate normobaric hypoxia (FiO_2_: 0.145) compared to normoxia elicits additional performance benefits and induces more favorable BLa responses.

## Materials and Methods

### Participants

The sample size was estimated using a power analysis software (G^*^power Version 3.1.9.6, Bonn University, Bonn, Germany) based on the mean effect (*d* = 1.32) of the within group improvement in mean power output for repeated 30-s Wingate efforts, as conducted by Puype et al. (2013) The power analysis resulted in a calculated total sample size of seven participants. However, we were only able to recruit six participants who met our selection criteria for each training group. Twelve male university sprinters (Age: 20.4 ± 1.2 years, Weight: 63.8 ± 4.9 kg, Stature: 172.4 ± 6.2 cm, 100-m personal best sprint time: 11.49 ± 0.47 s, weekly training volume: 8–12 h/wk) volunteered to participate after they provided written informed consent. None of the participants were acclimatized or recently exposed to altitude, and had any injuries. All tests and trainings were conducted during their preparation training phase (December to January). The study was conducted according to the Declaration of Helsinki, with the protocol approved by the state Research Ethics Committee at the University of Tokyo (No. 430-2).

### Design

This study used a single-blinded, randomized control design. All participants confirmed they were familiar with sprint cycling exercise before the experiment started. About 2-3 days before testing, participants first undertook a complete familiarization session where they performed two repetitions of 30-s Wingate sprint in normoxia. Participants then completed pre-tests that consisted of conducting the RW performance test (see below). They were randomly assigned to either a training in hypoxia (*n* = 6) or normoxia (*n* = 6) group that was based on the sum of total work achieved during pre-tests. We verified that there was no significant difference between hypoxic (652.6 ± 39.0 kJ/kg) and normoxic (672.5 ± 43.4 kJ/kg) training groups (*p* = 0.42). Two days after the pre-tests session, participants undertook six training sessions over 2 weeks (3 weekly sessions). Seven to nine days after the last training session, participants performed post-tests using the exact same procedure than pre-tests. All testing and training sessions were conducted in a temperature-controlled room (20°C) at the same time of day for each individual. Participants ingested their last meal 3-4 h before testing and were instructed to replicate the similar 24 h nutritional regimen between pre- and post-tests. Participants were instructed to refrain from taking caffeine and any other supplements, and avoid heavy exercise at least 24 h before testing/training sessions.

### Testing Procedures

Testing was conducted on a competitive-use road bike connected with a direct drive cycle trainer (T2800 NEO Smart Trainer, Tacx, Netherlands). Based on the manufacturer information, this cycle ergometer allows measurement of power output every second with an error of <1% (Tacx website, 2020). Seat and handle positions were reproduced for every session for each participant. Participants performed 3 × 30-s Wingate “all-out” efforts with 4.5 min of passive recovery. This was preceded by a standardized warm-up composed of 10-min low-intensity exercise (100 W, 90 rpm) followed by 2-3 × 6-s maximal sprints and finally a 5-min passive rest period before the first Wingate effort. During 6-s maximal sprints, participants confirmed their power outputs after each sprint and selected optimal gearing for Wingate efforts. Participants used same gearing between pre- and post-test, and between Wingate efforts during testing. They were asked to remain sitting during Wingate efforts. Peak power output (PPO), mean power output (MPO), total work for the three Wingate bouts, and the percentage decrement score were determined (Girard et al., 2011). A capillary blood sample was taken from the fingertip and analyzed for blood lactate (BLa) concentration with a portable analyzer (Lactate Pro 2, Arkray, Japan) immediately before the first Wingate sprint and 3 min after each bout. RPE (6–20 Borg scale) was collected 1 min after each exercise repetition.

### Training Procedure

Over 2 weeks, participants performed six RW training sessions (3 × 30-s Wingate “all-out” efforts with 4.5-min recovery) in either normobaric hypoxia (FiO_2_: 14.5%) or normoxia (FiO_2_: 20.9%). Participants were blinded to environmental conditions. They were fitted with a facemask fastened with a Velcro headset connected via plastic tubing to a hypoxic generator (YHS-B05, YKS, Japan) to simulate normobaric hypoxia using an oxygen-filtration technique. Participants started to inhale the hypoxic or normoxic air from the beginning of the warm-up (similar to testing procedures) until the termination of the last Wingate sprint when mask was removed (total exposure time was ~26.5 min in hypoxic condition). Training was conducted on the same competitive-use road bike and direct drive cycle trainer as for pre- and post-tests. Participants used same gearing as during pre-test for all training sessions. For each training session, MPO was measured to calculate total work completed during for whole intervention. All participants maintained their regular sprint-specific training (4 × 2-3 h training sessions per week) during the duration of the protocol. To avoid any effect of residual fatigue induced by prior exercise, all RW training sessions were performed either before sprint-specific training sessions (training days) or on “rest” days (no sprint-specific training). All participants were asked to continue same track and field training during the whole experimental period.

### Statistical Analysis

Two-way and three-way repeated-measures mixed-design ANOVAs were performed to compare experimental variables over Wingate efforts [Interval (before, bout 1, bout 2 vs. bout 3)] between the two groups [Group (hypoxia vs. normoxia)] and between before and after the training [Time (pre- vs. post-tests)] for both groups. To assess assumptions of variance, Mauchly's test of sphericity was performed using all ANOVA results. A Greenhouse–Geisser correction was applied to adjust the degree of freedom if an assumption was violated, while a Bonferroni *post-hoc* test for multiple comparisons was performed if a significant main effect was observed. Cohen's *d* values were calculated to examine the significance of the training improvements within and between each group. All values are expressed as mean ± standard deviation. Statistical significance was set at *p* < 0.05.

## Results

### Exercise Performance

**Figure 1:**
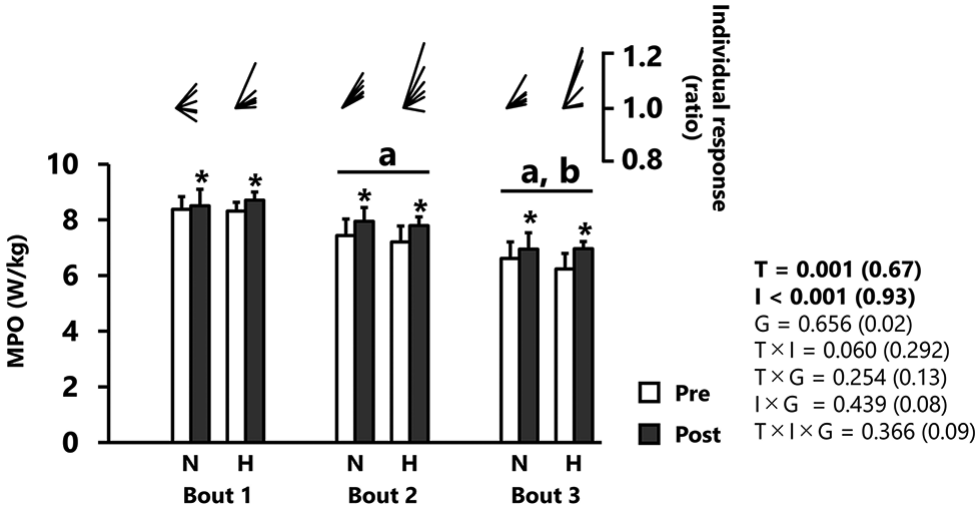
Mean power output (MPO). All values are expressed as mean with SD for error bars. Individual response is shown as a ratio of the value between pre and post within participants. Significance level and effect size are expressed as *P*-value (Cohen's *d*) for main effects and interactions. *p = 0.001 Compared with pre-test. ^a^*p* < 0.01 Compared with bout 1. ^b^*p* < 0.01 Compared with bout 2. N, normoxic group; H, hypoxic group; T, time; I, interval; G, group.

MPO significantly increased from pre- to post-tests (p < 0.001, *d* = 0.67), independently of group (Figure 1 and Table 1). MPO decreased after each successive sprint repetition (bout 1 vs. 2: −10.3 ± 5.9%; bout 2 *vs*. 3: −12.0 ± 3.4%; *p* < 0.001, *d* = 0.93), irrespectively, of group (Figure 1).

**Table 1:** Percent change for performance variables between pre- and post-tests.

	**MPO**			**PPO**			**Total work**	**Sdec**
	**Bout 1**	**Bout 2**	**Bout 3**	**Bout 1**	**Bout 2**	**Bout 3**		
Hypoxic group	+ 4.8 (10.3)	+ 8.6 (8.1)	+ 12.3 (10.1)	+ 9.2 (6.0)	+ 11.2 (6.9)	+ 20.7 (18.7)	+ 8.2 (7.1)	+ 17.6 (16.1)
Normoxic group	+ 1.6 (4.8)	+ 7.0 (2.9)	+ 5.2 (4.0)	+ 5.1 (10.2)	+ 4.9 (6.3)	+ 16.8 (24.0)	+ 4.4 (2.2)	+ 12.6 (26.2)
SMD	0.63	0.27	0.92	0.49	0.95	0.18	0.71	0.34

*All values are expressed as mean (standard deviation). Standardized mean difference (SMD) is expressed as cohen's d for percent changes between groups. MPO, Mean power output; PPO, Peak power output; Sdec, Sprint decrement score*.

**Figure 2:**
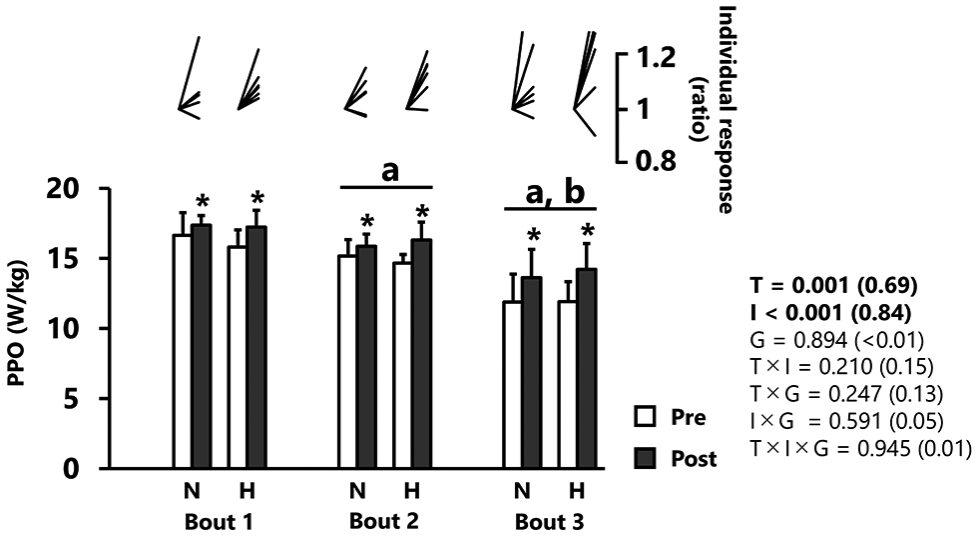
Peak power output (PPO). All values are expressed as mean with SD for error bars. Individual response is shown as a ratio of the value between pre and post within participants. Significance level and effect size are expressed as *P*-value (Cohen's *d*) for main effects and interactions. *p = 0.001 Compared with pre-test. ^a^*p* < 0.01 Compared with bout 1. ^b^*p* < 0.01 Compared with bout 2. N, normoxic group; H, hypoxic group; T, time; I, interval; G, group.

PPO significantly increased from pre- to post-tests (*p* < 0.001, *d* = 0.69), independently of group (Figure 2 and Table 1). PPO decreased after each successive sprint repetition (bout 1 vs. 2: −7.4 ± 4.8%; bout 2 *vs*. 3: −16.8 ± 10.3%; *p* < 0.001, *d* = 0.84), irrespectively, of group (Figure 2).

Total work was improved after vs. before the intervention, independently of group (+ 6.3 ± 5.4%; *p* = 0.016, *d* = 0.99, Table 1). Sdec did not differ (*p* = 0.11) between pre- and post-tests in either normoxic (12.6 ± 28.8%) or hypoxic (17.6 ± 17.7%) groups (Table 1).

Total work for six training sessions did not differ between normoxic (4,201 ± 240 kJ/kg) and hypoxic (3,992 ± 110 kJ/kg) groups (*p* = 0.13).

### Physiological and Perceptual Responses

**Figure 3:**
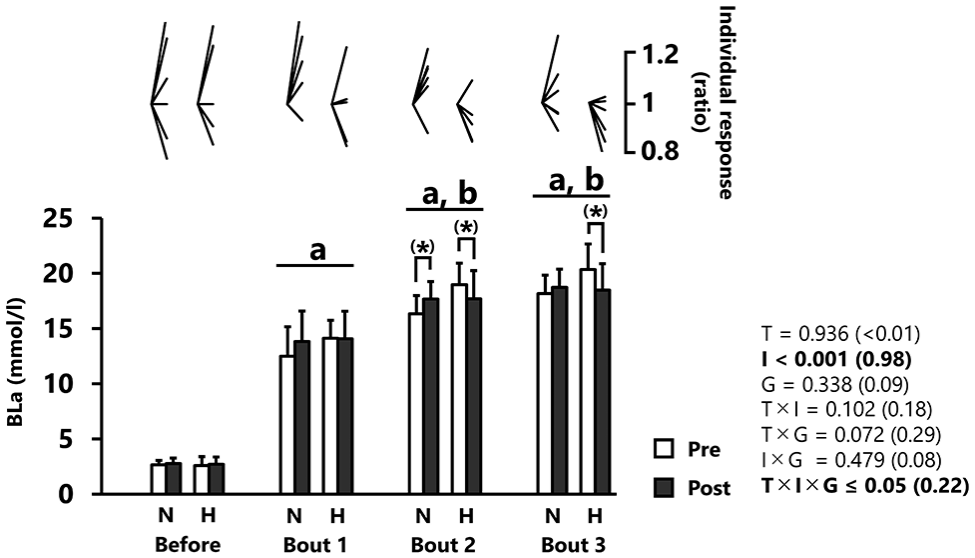
Blood lactate concentration (BLa). All values are expressed as mean with SD for error bars. Individual response is shown as a ratio of the value between pre and post within participants. Significance level and effect size are expressed as *P*-value (Cohen's *d*) for main effects and interactions. (*) 0.05 < *p* < 0.10 Tendency between pre- and post-test. ^a^*p* < 0.001 Compared with before. ^b^*p* < 0.001 Compared with bout 1. N, normoxic group; H, hypoxic group; T, time; I, interval; G, group.

BLa increased significantly across repetitions (*p* < 0.001, *d* = 0.98, Figure 3). *Post-hoc* analysis revealed elevated BLa values after bout 1 (13.6 ± 2.4 mmol/l) and even more so after bout 2 (17.7 ± 2.1 mmol/l) relative to before, but no difference between bouts 2 and 3 (18.9 ± 2.1 mmol/l). BLa increased (albeit not significantly) from pre- to post-tests after bout 2 in the normoxic group (+8.7 ± 10.3%; *p* = 0.08, *d* = 0.50, Figure 3). An opposite pattern was observed in the hypoxic group with lower values during post- compared to pre-tests after bout 2 (−9.3 ± 8.6%; *p* = 0.08, *d* = 0.67) and bout 3 (−9.1 ± 10.7%; *p* = 0.09, *d* = 0.81), although statistical significance was not reached (Figure 3).

**Figure 4:**
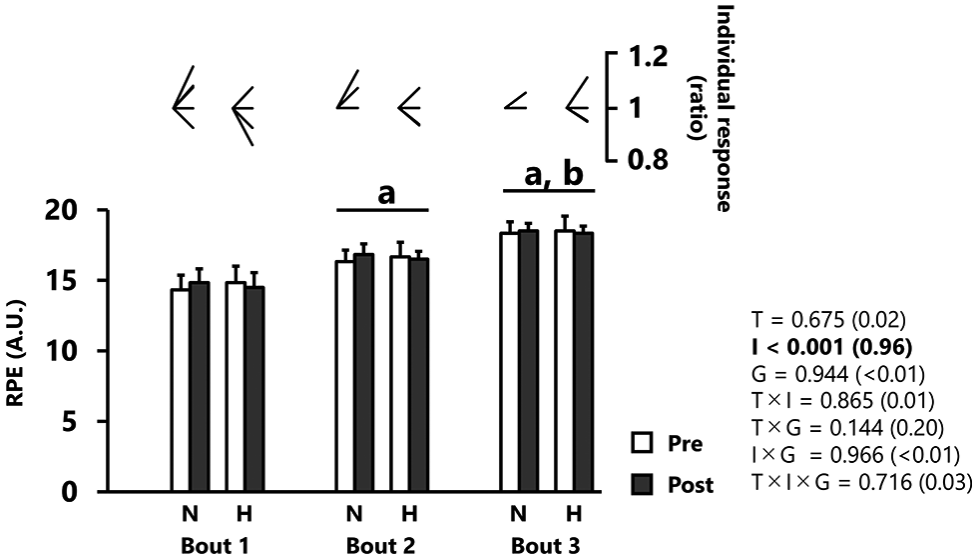
Rating of perceived exertion (RPE). All values are expressed as mean with SD for error bars. Individual response is shown as a ratio of the value between pre and post within participants. Significance level and effect size are expressed as *P*-value (Cohen's *d*) for main effects and interactions. ^a^*p* < 0.001 Compared with bout 1. ^b^*p* < 0.001 Compared with bout 2. N, normoxic group; H, hypoxic group; T, time; I, interval; G, group.

RPE was significantly increased after each successive repetition (*p* < 0.001, *d* = 0.96), independently of group (Figure 4). No main effect of training was reported for RPE values (*p* = 0.675)

Body weight did not differ (*p* = 0.99) between pre- and post-tests in both hypoxic (62.5 ± 3.6 vs. 62.6 ± 3.6 kg, *d* < 0.01) and normoxic (65.4 ± 4.9 vs. 65.3 ± 4.8 kg, *d* < < 0.01) training groups.

## Discussion

### Performance Outcomes

Contrary to our hypothesis, compared to normoxia, exposure to hypoxia during RW training failed to induce additional performance improvements. One previous study (Kon et al., 2015) and our preliminary data (not shown) suggest that acute moderate to severe hypoxia (FiO_2_ ranging from 16.4 to 13.6%) induces higher arterial deoxygenation but has no detrimental effects on RW exercise performance (3–4 × 30-s “all out” Wingate sprints with 4–4.5 min recovery) compared to normoxia. Accordingly, RW training in moderate to severe hypoxia likely induces higher physiological stress (lower oxygen flux) for a similar mechanical stimulus. In our study, however, adding hypoxic exposure to RW training had no performance benefit. One possible explanation may reside in the relatively small number of participants that would prevent detection of significant differences. Despite relative gains in MPO and PPO for each of the three bouts being larger in the hypoxic compared to the normoxic training group (Table 1), large inter-individual variability likely obscured results of statistical analysis. In the current study, we only recruited trained sprinters rather than recreationally active participants to increase the practical relevance of our findings. Because the number of participants who were fitting our inclusion criteria was limited, our observations would need to be confirmed in future studies with larger sample sizes.

In this study, six RW training sessions conducted over 2 weeks induced significant improvements in all performance indices (higher MPO, PPO and total work with unchanged sprint decrement scores) in trained sprinters, independently of condition. Although training load and hypoxic dose were rather small training load and hypoxic dose (a total of 9 min of effort at ~3,000 m simulated altitude), from a practical point of view, RW training induced robust performance improvements in already well-trained participants. Previous studies also reported comparable performance gains (MPO: +9.5–10.1%; PPO: +10.6–12.1%) after 6 sessions of normoxic RW training in sedentary or recreational level participants (Gist et al., 2014). Similar observations have been made using the same number of RW training sessions (+2.4% in MPO for the best of 4 bouts of 30-s “all out” exercise) in endurance trained subjects (Koral et al., 2018). Compared to this later study, we observed similar degrees of MPO improvement (+1.6 ± 4.8% for normoxic and +4.8 ± 5.4% for hypoxic group) in well-trained participants using same number of RW training sessions. Therefore, in already trained participants, six RW training session can still induce significant performance benefits.

In the current study, performance improvement was measured 7–9 days after completing the training intervention. One previous study reported that performance during a single Wingate effort improved when assessed 9 days but not 2 days after the hypoxic training intervention (Hendriksen and Meeuwsen, 2003). Two other studies using trained sprinters reported that 5 to 6 consecutive days of hypoxic training composed of RW (repeated maximal sprints of 15–30 s) and repeated sprint training (repeated maximal 6-s sprints) failed to induce improvement in single Wingate effort 3 days after the intervention (Kasai et al., 2017, 2019). Since performance was not assessed in the first few days or several weeks after the intervention, immediate and long-term consequences of our intervention are unknown. While it cannot be ascertained that performance gains would have reached a peak at this time, our data support a view that 7–9 days would allow sufficient recovery from the intervention to measure positive effects. In the real world, most athletes would normally train hard up to 1-2 weeks prior to the actual competition, and then taper their training in the days preceding major events (Mujika and Padilla, 2003). With this in mind, our training routine seems practically relevant to be included in the busy schedule of sprinters in the lead up of competition.

### Physiological Adaptations

BLa concentration values measured 3 min after each sprint (ranging ~14–20 mmol/l) increased across successive intervals (yet with no statistical difference between bouts 2 and 3), independently of group or time. This indicates that our RW exercise relied heavily on glycolytic energy production in the initial two bouts, but that mitochondrial energy production might play an increasingly role to satisfy energy demand during the last effort. These observations are in line with a previous study in which glycolytic energy production was greater and oxidative enzyme activity was not fully activated in the first bout, while an opposite pattern was observed in the last bout of 3 × 30-s Wingate “all-out” efforts with 4 min recovery (Parolin et al., 1999).

There was a significant time × group × interval interaction for BLa. In the normoxic group, BLa concentration in response to bout 2 tended to increase from pre- to post-training. It is a common observation that sprint interval training in normoxia increases post-exercise BLa concentration (Sharp et al., 1986; Rodas et al., 2000; Creer et al., 2004; Bayati et al., 2011). This may relate to an increased glycolytic enzyme activity, as demonstrated elsewhere (MacDougall et al., 1998; Rodas et al., 2000). In our study, it is possible that RW training in normoxia increased glycolytic enzyme activity. In the hypoxic group, BLa concentration following bouts 2 and 3 tended to be lower after compared to before the intervention. Contrastingly, one previous study reported that RW training in hypoxia compared to normoxia significantly increased glycolytic enzyme activity (Puype et al., 2013). In line with our results, however, we (Takei et al., 2020b) and others (Gatterer et al., 2018) have also reported lower BLa after training in hypoxia. In trained cyclists, we recently observed that hypoxic exposure induced a ~9% decrease in BLa concentration values (10-min area under the curve) after a single Wingate effort following six identical RW training sessions than those performed here (Takei et al., 2020b). Furthermore, Gatterer et al. (2018) observed that 9 sessions of hypoxic RW training (4 × 30-s Wingate “all-out” efforts with 5-min recovery) induced a ~17% decrease in BLa from pre- to post-training measured 3 min after a single Wingate effort. several limitations may preclude firm conclusions to be drawn in the study by Gatterer et al. (2018). First, observation of lower BLa concentration values with hypoxic training did not reach statistical difference, possibly due to a small sample size (*n* = 5). Second, no normoxic training group was included in their pilot study, making it difficult to determine the true effect of hypoxic exposure. While our study was designed to overcome this later limitation, the reader must be cognizant that our sample of highly trained runners (*n* = 6 for each group) also was rather small. Post-exercise BLa concentration can be influenced by both lactate production and oxidation (Brooks, 2018). Reportedly, endurance training in hypoxia can increase total and subsarcolemmal (i.e., those located near capillaries) mitochondria volume compared to similar training in normoxia, while these observations are not specific to RW training (Hoppeler et al., 2003). Collectively, our BLa results would indirectly suggest that adding hypoxic exposure to RW training boosts mitochondrial adaptations, in turn increasing lactate oxidation.

### Additional Considerations and Limitations

RW training is known to preferentially induce peripheral (skeletal muscle arterial-venous oxygen difference) rather than central (cardiac output) adaptations (Macpherson et al., 2011). Moreover, hypoxic training is more likely to evoke non-hematological (including changes in enzyme activities, mitochondrial density and capillarization) compared to hematological adaptations. Therefore, although we have not included muscle oxygenation data (as indirectly assessed with near-infrared spectroscopy), it is possible that RW training in hypoxia induced larger improvement in oxygen utilization in active musculature. In support, larger improvement in oxygen utilization was observed in trained cyclists (Faiss et al., 2013a) and cross-country skiers (Faiss et al., 2015) who performed repeated sprints training (5 × 10-s “all-out” sprints) in hypoxia than normoxia. This suggestion is indirectly supported by observation of lower BLa concentrations during RW exercise in hypoxia. Future studies examining muscle oxygenation trends are needed to shed some light on our assumption that hypoxia facilitates an increased rate of lactate oxidation.

Another consideration when interpreting our results is the severity of hypoxic condition. Arguably, too severe hypoxic levels would decrease absolute exercise intensity and/or volume, in turn impairing the overall training stimulus. In order to overcome this possible limitation, one recent work applied severe hypoxia (FiO_2_: 0.106–0.114) during exercise periods of a high intensity interval training routine (80–85% of maximal aerobic speed), while recoveries were conducted in normoxia (Sanchez and Borrani, 2018). Using this training paradigm, additional aerobic and anaerobic performance benefits were reported in highly trained endurance athletes, compared to training with exercise and subsequent recovery segments in normoxia (Sanchez and Borrani, 2018). Future research may also verify if applying more severe hypoxia levels during the actual RW exercise (likely inducing more intense physiological stress), but normoxic recoveries (to preserve absolute training intensity and/or volume), is a useful approach.

A majority of previous RW training studies have used endurance athletes who typically performed four or more RW efforts during their training sessions (Creer et al., 2004; Koral et al., 2018). Here, we included only 3 exercise bouts of 30 s with 4.5-min recovery because sprinters (likely less resistant to fatigue) were recruited. Previous studies also including 3 RW efforts reported significant performance benefits (increased MPO and PPO) and physiological adaptations (increased number of type IIa fibers) in sedentary and active participants (Allemeier et al., 1994; Ijichi et al., 2015). To our knowledge, no previous study examined whether 3 RW represents an optimal number of repetitions to maximize performance benefits in trained participants. Perhaps more desirable outcomes would be obtained if a larger number of repetitions and/or a modification of the exercise-to-rest ratio (especially when exposed to hypoxia to preserve training quality) was adopted.

In this study, we used repeated sprint interval training in hypoxia on a cycle-ergometer for sprint runners who usually compete in 100- and 200-m events. Although 100- or 200-m race times are clearly shorter than 30 s, it is common for these athletes to perform workouts of this duration during their preparation phase. Practically, one study showed that 6- to 20-s repeated sprint cycling training in hypoxia improved 60-m sprinting performance in 100–200 m sprint runners, with these effects being mainly visible for the 0–10 m distance interval (Kasai et al., 2019). Moreover, efforts were performed on a cycle ergometer during this period of training in order to minimize ground contacts and the possibly of sustaining an injury since 6 sessions were planned over a 2-wks period.

While the two groups performed the same amount of total work across the 6 RW training sessions, training load for track and field workouts was not specifically quantified. Therefore, we cannot deny the possibility that loads during track and field training sessions might differ slightly between groups. That said, all participants were belonging to the same track and field club, trained as a group and were following a similar weekly training schedule.

Another limitation of this study lies with the capacity of the hypoxic generator. This device can produce ~80 L/min of hypoxic air, which would match the average demand of ventilation (~60 L/min) during repeated Wingate efforts, as reported elsewhere (Freese et al., 2013). However, it is also known that peak ventilation rate typically ranges between 100 and 120 L/min at the end of each effort (Oguri et al., 2008; Freese et al., 2013). Although it was not measured, in our study, the time spent at a ventilation rate exceeding 80 L/min was probably limited (~4 min out of a total of ~26.5 min being exposed to hypoxia). Nonetheless, it cannot be totally ruled out that the effects of hypoxia were slightly underestimated.

## Conclusion

Six sessions of RW training (3 × 30-s Wingate “all-out” efforts with 4.5-min recovery) performed 3 times per week over 2 weeks in either hypoxia or normoxia led to similar performance gains. However, with the addition of hypoxia BLa responses became more favorable, indicating that physiological adaptations (increased lactate oxidation) with such exercise intervention may depend on oxygen availability.

## Data Availability Statement

The datasets generated for this study are available on request to the corresponding author.

## Ethics Statement

The studies involving human participants were reviewed and approved by Research Ethics Committee at the University of Tokyo. The patients/participants provided their written informed consent to participate in this study.

## Author Contributions

NT, KK, and HH: experiment design. NT and KK: experiment implementation. NT, KK, and OG: data analysis. NT, OG, and HH: paper composition. KK, OG, and HH: analyzing and writing advisory. All authors contributed to manuscript revision, read, and approved the submitted version.

## Conflict of Interest

KK, an employee of Blue Wych Limited Company, voluntarily participated in the present study. We declare that there is no conflict of interests regarding the publication of this article. The remaining authors declare that the research was conducted in the absence of any commercial or financial relationships that could be construed as a potential conflict of interest.
